# A global dataset on mungbean for managing seed yield and quality

**DOI:** 10.1038/s41597-025-05016-6

**Published:** 2025-04-19

**Authors:** Natalia da Silva Volpato, Federico M. Gomez, Víctor D. Giménez, Ignacio A. Ciampitti

**Affiliations:** 1 Department of Agronomy, Kansas State University, Manhattan, Kansas US; 2 Department of Agronomy, Purdue University, West Lafayette, Indiana US

**Keywords:** Agriculture, Developing world

## Abstract

Mungbean, a protein- and nutrient-rich legume widely grown in Asian countries, is now expanding to other regions. With increasing global demand for nutritious foods and more sustainable agricultural practices, mungbean represents a valuable opportunity to diversify food systems and improve global nutrition. However, management practices to optimize seed yield and quality (including seed oil and protein concentration) remain largely unexplored due to the recent introduction of this crop in many parts of the world. This study aims to create a global open dataset of key crop management practices and their impact on seed yield parameters and quality. This dataset included 227 studies published between 1984 and 2023, comprising 2,154 observations. It focuses on key agronomic practices, including fertilization, irrigation, row spacing, seeding rate, and planting date. By providing comprehensive insights into mungbean cultivation, this open-access dataset enables researchers and agronomists to optimize management practices, identify research gaps, and enhance seed yield and nutritional quality. It serves as a valuable tool for improving food security and sustainable agriculture.

## Background & Summary

Mungbean [*Vigna radiata* (L.) R. Wilczek] is a summer crop legume, cultivated on about 7 Mha worldwide^1^. Today, nearly 90% of the mungbean production is found in Asia, where India, China, Pakistan, and Thailand are among the most important producers, but mungbean is also produced in parts of Africa, Australia, North and South America^2–4^. This crop has many advantages that make it a good option for inclusion in crop rotations or intercropping systems. It has a short growth cycle (70–95 days), with low-input requirements, and as a legume, it fixes most of its nitrogen^5^ allowing growers to increase profitability and sustainability of their farms. Mungbean also plays an important role in nutritional feed. The seeds are rich in easily digestible protein (~24%), fiber, antioxidants, and phytonutrients, and they can be consumed whole, split, ground into flour, or used as sprouts^6^. This nutritional profile makes mungbean an excellent addition to a balanced diet with cereals, with this legume as a less expensive available source of protein for developing countries^7^ and for vegetarians. However, mungbean yields are low on a global scale, averaging 0.73 t ha^−1^, with a great potential to for enhancement^8^.

Understanding the impact of different agronomic practices on mungbean is crucial for developing strategies that enhance both seed yield and quality and reduce seed yield variability. A comprehensive global dataset offers a unique opportunity to analyze the variability in mungbean performance under diverse environmental conditions. This global dataset can help researchers identify high-performing management practices that lead to increased seed yield and quality or reduce seed yield variability. In addition, this global data set provides two important benefits. First, it enables direct comparisons of a wide range of agronomic practices, helping to identify those most effective in different contexts. Second, it allows researchers to assess current knowledge of practices that affect mungbean yield components and quality, and to identify knowledge gaps in existing research. By highlighting areas where future research is needed, this dataset can guide efforts to further optimize mungbean production. Lastly, by making this dataset available, it supports the broader scientific community in its efforts to evaluate and improve the agronomic and environmental performance of this cash crop.

## Methods

### Data collection

A comprehensive literature search was conducted in March 2024 across several databases, including Wiley, ScienceDirect, Scopus, and Web of Science, followed by a subsequent search in March 2025 using Google Scholar, to compile data on mungbean cultivation. The search equation was formulated to include key terms related to the crop and its management practices, focusing on the title, abstract, or keywords. The terms used were (“Mungbean” or “Mung bean” or “Vigna radiata” or “Green gram”) and (“yield” or “protein” or “oil”) and (“harvest” or “fertilization” or “watering” or “plant density” or “planting date” or “sowing date” or “irrigation” or “row spacing” or “seeding rate” or “fertilizer rate”). The studies on other species such as Black gram or Vigna mungo were explicitly excluded.

**Fig. 1:**
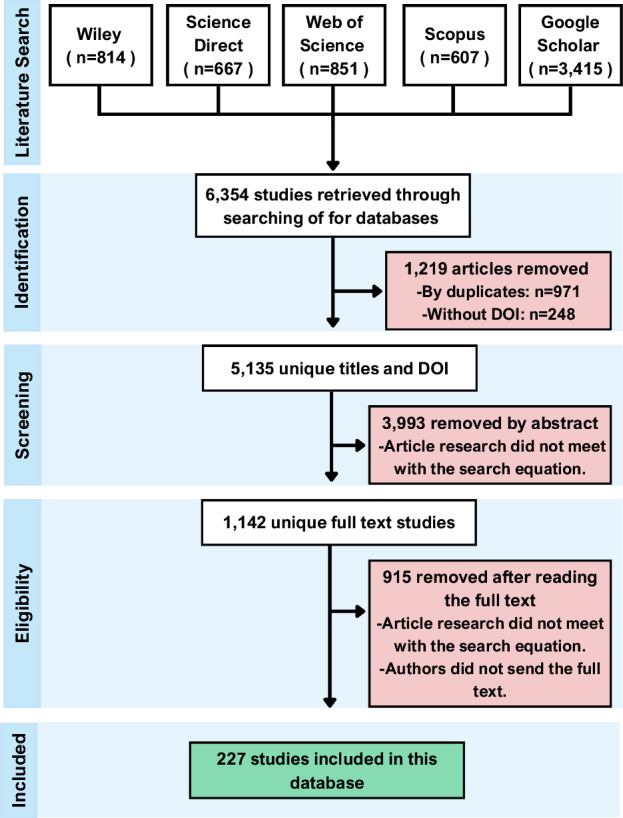
PRISMA workflow diagram for the systematic dataset construction, showing the different steps and the number of publications in each step (n).

For the publisher Wiley, the search was restricted to articles within the field of agriculture, resulting in 814 publications. For ScienceDirect, the search was limited to agricultural and biological sciences, including review articles, research articles, and short communications, yielding a total of 667 publications. The search in Scopus, confined to agricultural and biological sciences and English-language articles, reviews, and journals, resulting in 607 publications. For Web of Science, the search was restricted to areas of Agriculture multidisciplinary, Plant Science agronomy, or Food science and technology, including Articles, early access publications, and Proceedings Papers, also limited to English, resulting in a total of 851 publications. Finally, the bibliographic search was conducted in Google Scholar using the Publish or Perish software^9^. The search equation was employed to search the 2,000 most relevant publications from 1984 to 2025 by title and then by keywords, resulting in a total of 3,415 effective publications. In summary, the initial search across all search engines produced a total of 6,354 published articles (Fig. 1).

The first screening step involved removing duplicates and articles without DOI, reducing the dataset to 5,135 publications (Fig. 1). A subsequent abstract screening was conducted to eliminate studies that did not address the specific topics outlined in the search equation, resulting in 1,142 relevant articles. The process of identifying duplicates and conducting the abstract screening was facilitated by the R package devtools^10^.

The final screening involved a detailed manual review of the full articles to ensure they reported the effects of key management practices defined in the search equation- on seed yield, protein, and oil concentrations in mungbean. Only studies conducted under field conditions were included, leading to the selection of 227 publications comprising a total of 2,154 individual observations (Fig. 1).

Data extracted from these articles included plant growth parameters, yield, and seed components, and were recorded manually. In cases where data were presented graphically, figures were digitized using WebPlotDigitizer (https://automeris.io/WebPlotDigitizer). In addition, relevant metadata were collected, such as authorship, year of publication, experimental design, information on laboratory methods for determining seed components, soil characteristics (soil type, texture, and pH), and details about fertilizer application (timing, type), were also gathered to provide context for the observations.

Additionally, annual mean temperature and total precipitation were collected from the studies when reported by the authors. To supplement the weather data, we also included annual mean temperature, total precipitation, annual mean humidity, and total solar radiation from NASA Power^11^ for studies that did not report weather data, as well as for those that did. Thus, the weather data in this database include both observations from the original studies and additional data from NASA Power.

## Data Records

The dataset generated from this study is publicly accessible through the Figshare repository^12^, available at 10.6084/m9.figshare.27111106. The repository contains the following files:

1. “Mungbean_dataset.xlsx”: This file includes the comprehensive global dataset.
2. “Metadata.docx”: This document provides a detailed explanation of the dataset, describing each column and the units of measurement for each variable.
3. “References.docx”: A compilation of all the references from the articles used in this study.
4. “Code.zip”: The scripts used to generate the figures presented in this study.

The “Mungbean_dataset.xlsx” can be divided into three key sections:

- First Section: Contains publication-specific details, such as the paper ID, authors, year of publication, URLs, and DOI for each article.
- Second Section: Focuses on experimental design and field-related information. This includes the study coordinates, location, year of study, experimental design, number of replications, and descriptors (main, secondary, and additional).
- Third Section: Contains the plant growth, yield, and seed quality parameters. This includes metrics such as plant weight, number of leaves per plant, number of branches, number of pods per plant, pod length, number of seeds per pod, seed weight, total seed yield, biological yield (total dry matter produced per plant or unit area), and harvest index (ratio of seed to total above-ground dry matter). Seed components such as protein, oil, starch, and carbohydrates, and the laboratory methods used to determine these components, are also included.

Additionally, the dataset captures relevant soil characteristics, including soil texture, type, pH, bulk density, organic carbon content, and the depth at which these parameters were measured. Weather data, including annual mean temperature, total precipitation, annual mean humidity, and total solar radiation were also recorded. Furthermore, fertilizer-related information was collected, including fertilizer type, time of application, and key nutrients.

**Fig. 2:**
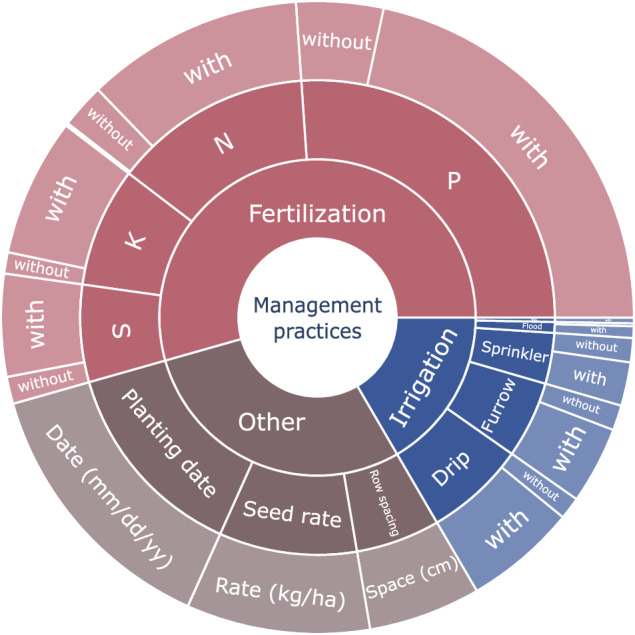
Sunburst Diagram of the complete hierarchical structure. At the center, the first layer of information includes the main descriptors, followed by a second layer of information, the secondary descriptors, and lastly, on the outside is the third layer of information, the additional descriptors. The area of each region is proportional to the number of observations included in each section of the database.

The treatments applied in the publications were classified into three main descriptors in the dataset: ‘Fertilization’, ‘Irrigation’, and ‘Other management practices’ (Fig. 2). The main descriptor corresponds to the management focused on the search theme defined by the authors. ‘Fertilization’ refers to those treatments in which different fertilizer treatments were evaluated. ‘Irrigation’, corresponds to those treatments in which the effect of irrigation was evaluated. ‘Other’ refers to those treatments in which different treatments for planting date, seeding rate, and row spacing were evaluated.

These descriptors are further divided into subcategories (secondary descriptors):

- Fertilization was divided into nitrogen (N), phosphorus (P), potassium (K), and sulfur (S) treatments.
- Irrigation subcategories include sprinkler, furrow, flood, drip, and wick irrigation methods.
- Other management practices were divided into planting date, seeding rate, and row spacing.

‘Nitrogen’, ‘Potassium’, ‘Phosphorus’, and ‘Sulfur’ (N, P, K, and S) are the secondary descriptors for Fertilization, and refer to treatments in which nitrogen, potassium, phosphorus, and sulfur respectively were classified as a main nutrient. If more than one nutrient was applied in the treatment, it was classified according to the nutrient with the highest concentration in the formulation.

‘Sprinkler, furrow, flood, drip, or wick’ subcategories, in the secondary descriptor for ‘Irrigation’, correspond to different types of irrigation systems described in each article. The subcategories ‘Planting date’, ‘Seeding rate’, and ‘Row spacing’ into ‘Other management practices’ refer to management practices associated with treatments of the same name.

For each secondary descriptor, sub-subcategories, known as **‘**Additional descriptors’, were used:

- ‘N, P, K, and S’ were divided into ‘With fertilization’ and ‘Without fertilization’. In addition, the reported rate of nutrients applied was included along with this classification.
- ‘Sprinkler’, ‘Furrow’, ‘Flood’, ‘Drip’, and ‘Wick’ were divided into ‘With irrigation’ and ‘Without irrigation’. In addition, the reported amount of water applied, or the timing of application was included along with this classification.
- ‘Planting date’, ‘Row spacing’, and ‘Seeding rate’ were divided into the date (mm/dd/yy), space (cm), and rate (kg seed ha^−1^), respectively.

The complete classification (Main, Secondary, and Additional descriptors) is presented in Fig. 2 to provide a comprehensive overview of the study design and management practices used across the included articles.

## Overview of the Dataset

**Fig. 3:**
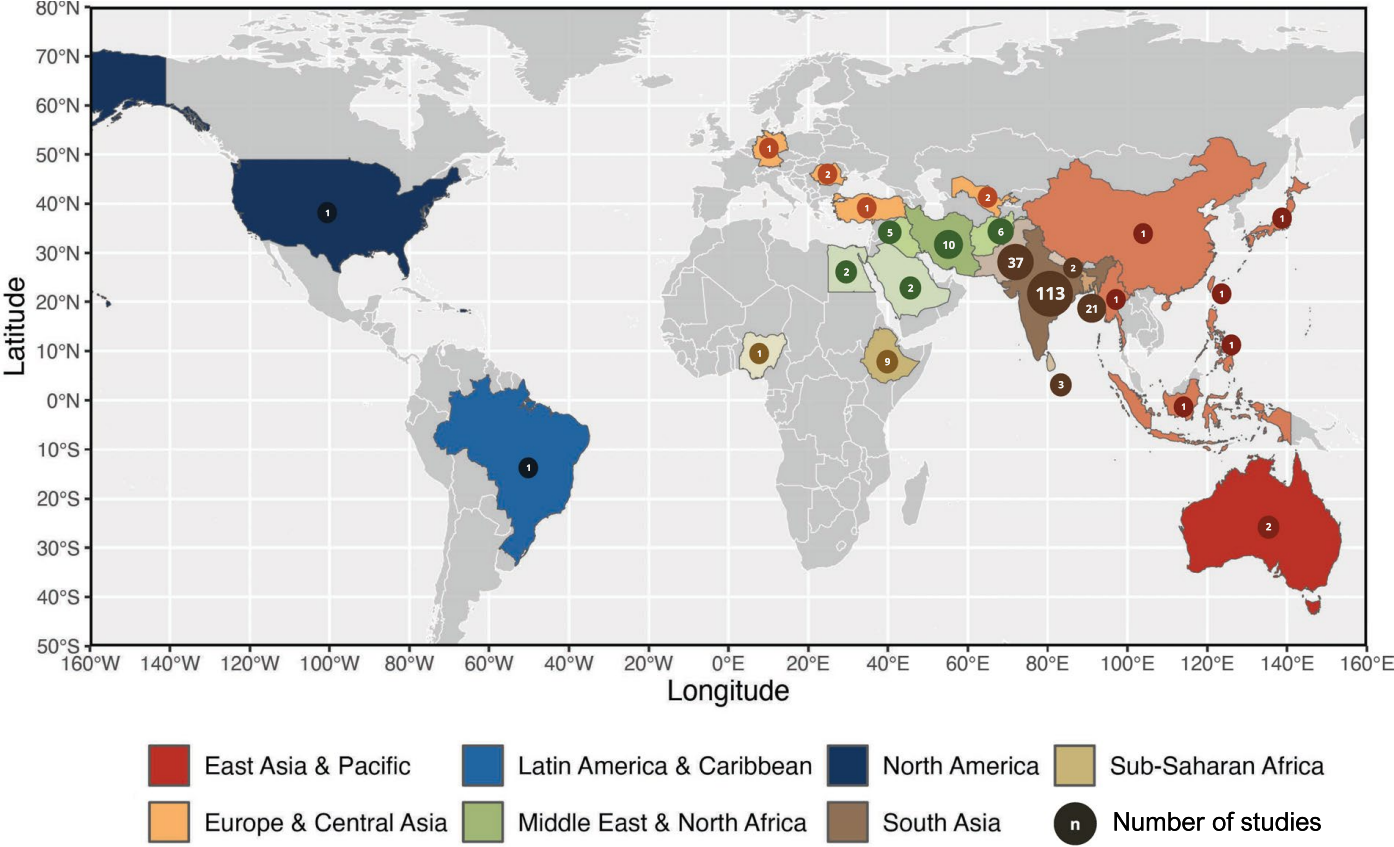
Global distribution of the studies included in the database. The number within the bubble and the size of the bubble represent the total number of studies synthesized and included in the open dataset for each country.

This dataset contains information extracted from papers published between 1984 and 2023 period. Most of these publications originated from countries in South Asia, Middle East, and North Africa, regions most frequently represented in our search criteria (Fig. 3). The most studied management practices were fertilization and irrigation (together accounting for ~73% of observations).

## Technical Validation

**Fig. 4:**
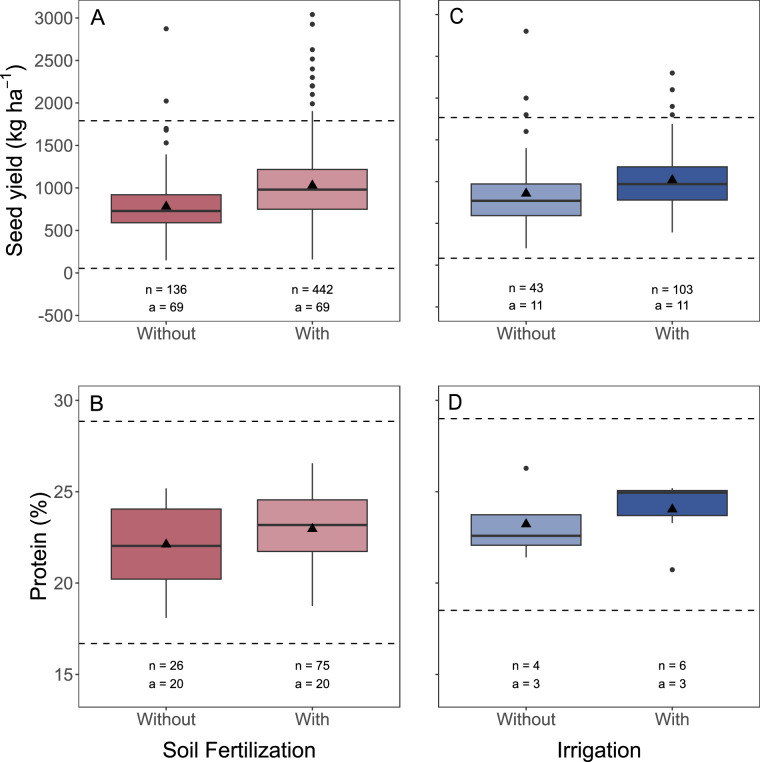
Box-plot of seed yield (panels A and C), and seed protein concentration (panels B and D) for Fertilization and Irrigation main descriptors. The solid black line represents the median. The edges of the box correspond to the first (Q1) and third (Q3) quartiles, while the difference between Q1 and Q3 represents the interquartile range (IQR). The whiskers indicate the minimum and maximum value (i.e., Q1 - 1.5 * IQR and Q3 + 1.5 * IQR). The dots correspond to potential outliers. The triangles correspond to the mean values of seed yield and protein concentration for each level within the treatments. The dashed horizontal lines represent the lower (i.e., Q1 - 1.5 * IQR) and upper (i.e., Q3 + 1.5 * IQR) limits for mild outliers. ‘n’ indicates the number of observations, and ‘a’ indicates the number of studies.

To assess the reliability of the retrieved dataset, a series of quality control measures were conducted. These included visual inspection, box-plot analysis, and detection of potential outliers for the main variables: seed yield and protein concentration (Fig. 4). The distribution of these variables was analyzed using the first quartile (Q1, the 25th percentile) and the third quartile (Q3, the 75th percentile). The interquartile range (IQR) was calculated as the difference between Q1 and Q3. Seed yield and protein concentration values below the lower threshold (Q1 - 1.5 * IQR) or above the upper threshold (Q3 + 1.5 * IQR) were identified as mild outliers. Additionally, extreme outliers were determined as those values falling below Q1 - 3 * IQR or above Q3 + 3 * IQR^13^.

Six mild outliers were identified in seed yield for fertilization, exceeding the upper threshold of 1,790 kg ha^−1^ (these observations were collected from IDs 7, 49, 53, 63, 139 and 169). Furthermore, five extreme outliers were identified in seed yield, with values exceeding 2,441 kg ha^−1^ (from IDs 80 and 139). Seven mild outliers were detected in seed yield for irrigation, with four observations exceeding the upper threshold of 1,768 kg ha^−1^ (corresponding to IDs 26, and 46). Only one extreme outlier was identified, with a value of 2,800 kg ha^−1^ (from ID 46). No mild or extreme outliers were observed in seed protein concentration for either fertilization or irrigation.

## Usage Notes

This open global dataset is an essential resource for researchers, agronomists, and stakeholders focusing on enhancing mungbean production. This global dataset provides detailed data on seed yield, protein concentration, and various plant growth parameters under different management practices influencing mungbean cultivation. Additionally, this open global dataset can serve to identify scientific knowledge gaps in existing research on key management factors for improving mungbean production and reducing its variability. Addressing these research gaps is critical for optimizing mungbean production and global food security.

## Data Availability

The global dataset, along with metadata, references, and the R software scripts, are accessible on the Figshare repository^12^.
